# Efficacy and safety of basic fibroblast growth factor in the treatment of burns: Protocol for a systematic review and meta-analysis of randomized controlled trials

**DOI:** 10.1097/MD.0000000000015102

**Published:** 2019-04-05

**Authors:** Da-Chuan Zhan, Yong-Shuai Shen, Yue-Rong Zhao, Fan-Jun Meng

**Affiliations:** Department of Burn and Plastic Surgery, Chifeng Municipal Hospital (Chifeng Clinical Medical School of Inner Mongolia Medical University), Chifeng, Inner Mongolia Autonomous Region, China.

**Keywords:** basic fibroblast growth factor, burn, meta-analysis, protocol, randomized controlled trial, safety, therapeutic effect

## Abstract

**Background:**

To systematically evaluate the efficacy and safety of basic fibroblast growth factor (bFGF) in the treatment of burns and to provide evidence-based medical information for clinicians to choose the appropriate treatment measures for burns.

**Methods:**

Seven databases, including PubMed, the Cochrane Library, Embase, the Chinese Biomedical Literature Database, the Wanfang Database, the China National Knowledge Infrastructure Internet, and the Chongqing Chongqing Weipu Chinese Science and Technology Journal Full-text Database (VIP), were searched by computer. Randomized controlled trials on bFGF in the treatment of burns were collected, and the search was conducted by using a combination of subject terms (MeSH) and free words. The search time limit was from the establishment of each database until January 2019. Two researchers independently screened the literature and extracted the data. According to the evaluation criteria recommended in the Cochrane Handbook for Systematic Reviews of Interventions version 5.3.0, they conducted a rigorous bias risk assessment for the included studies, and Stata 12.0 software was used for meta-analysis.

**Results:**

System evaluation and meta-analysis were carried out strictly in accordance with the requirements of the Cochrane Handbook for Systematic Reviews of Interventions version 5.3.0 on meta-analysis and provided a high-quality evaluation of the efficacy and safety of bFGF in the treatment of burns.

**Conclusion:**

This study provided conclusions from evidence-based medicine and a scientific basis for the efficacy and safety of bFGF in the clinical treatment of burns.

**Ethics and dissemination:**

This study was not a clinical trial and therefore did not require ethical approval. The results of this study will be published in an SCI academic journal related to this study in the form of a public publication.

**PROSPERO registration number:**

CRD42019124778.

## 1 Introduction

Burns are some of the most lethal, mutilating, disfiguring, and serious clinical infectious diseases. They are also some of the most commonly observed traumatic diseases. Burns are caused by the body's direct contact with high-temperature objects or by intense thermal radiation; external injuries such as burns, scalds, electrical injuries, and chemical burns of the skin or muscle are caused by flame, hot water, hot oil, electrical shock, or chemical irritants.^[1]^ When the body's natural barrier, the skin, is damaged by thermal energy, a series of changes in the body's internal environment cause the exudation of protein molecules^[2]^, and a large area of tissue is without protective coverage such that the burn wound is very vulnerable to infection caused by bacteria and other microorganisms.^[3]^ Therefore, promoting the healing of burn wounds has become one of the key points in the treatment of burns.

At present, there is no uniform definition for the concept of residual wounds in the late stages of a burn. Residual wounds usually refer to deep burn wounds that cannot heal in a short period of time (usually more than 4 weeks after injury), granulation wounds that have not healed in the skin graft space, granulation wounds that have not been operated on and wounds that have newly healed after bursting. The granulation tissues of these wounds are generally old and have no blood flow or edema. The healing time is longer, or the wound may even not heal by routine dressing change alone. It is easy for burn wounds to become infected, and erosions can fuse into tablets.^[4]^ Burn wound healing is a complex biological process that includes a series of cross cascade reactions, such as inflammation, cell proliferation, migration, and wound remodeling.^[5]^ Since Cohen discovered epidermal growth factor in 1962, more than a dozen growth factors have been found that are involved and regulated in all stages of wound healing.^[6]^

For the above reasons, choosing appropriate drugs to promote wound healing is an important part of tissue repair. With the development of modern molecular biology and biochemistry, people's understanding of the regulation of wound healing is deepening as well. Currently, the most common clinically applied growth factor is basic fibroblast growth factor (bFGF), which is a single chain polypeptide with an isoelectric point of 5.6. It is present in a cationic form under physiological conditions and contains amino acid residues. After enzymatic hydrolysis, active protein polypeptides are produced that can promote, repair and regenerate mesodermal and ectodermal cells such as epithelial cells, dermal cells, fibroblasts, and vascular endothelial cells.^[7,8]^ Although bFGF has been widely used in the clinic, there is still a lack of evidence-based medicine. To objectively evaluate the efficacy and safety of bFGF in the treatment of burns, a meta-analysis of randomized controlled trials in which bFGF was used in the treatment of burns at home and abroad was conducted to provide evidence for the use of bFGF in the treatment of burns.

## 2 Objectives

The purpose of this systematic review was to systematically evaluate the efficacy and safety of bFGF in the treatment of burns and to provide medical evidence to assist clinicians in choosing appropriate treatment measures for burns.

## 3 Methods and analysis

### 3.1 PROSPERO registration

Our systematic review and meta-analysis protocol was fully implemented in accordance with the requirements of the Preferred Reporting Requirements for Systematic Review and Meta-analysis Protocol (PRISMA-P) statement and the Cochrane Handbook for Systematic Reviews of Interventions. The system evaluation has been registered on the PROSPERO website, and the registration number is CRD42019124778.

### 3.2 Study inclusion and exclusion criteria

#### 3.2.1 Types of studies

A randomized, controlled trial of the use of bFGF in the treatment of burns was carried out. Regardless of whether a blind method or allocation hiding was used, there was no limit to the language used.

#### 3.2.2 Types of participants

##### 3.2.2.1 Inclusion criteria

The inclusion criteria were as follows:

(1) randomized controlled trials;
(2) hospitalized patients with burns ranging from 30% to 80% within 1 week of injury;
(3) burn depth diagnosed by the “three-degree-four-point method”^[9]^; burn area estimated by the “Chinese nine-point method”^[10]^;
(4) no statistically significant difference in the basic characteristics of patients in the treatment group and the control group, and the 2 groups were comparable; and
(5) no history of other medications used to treat burn wounds.

##### 3.2.2.2 Exclusion criteria

The exclusion criteria were as follows:

(1) non-RCT literature;
(2) incomparable basic characteristics of patients in the treatment group and the control group;
(3) animal experiments;
(4) case reports;
(5) imperfect literature reviews and conference papers reporting data with unresponsive associated authors;
(6) nonconformity to the pre-established selection criteria for treatment measures; and
(7) repeated publications.

#### 3.2.3 Types of interventions

##### 3.2.3.1 Experimental group

Patients in the experimental group received bFGF (similar dose, times of administration and course of treatment) plus routine treatment.

##### 3.2.3.2 Control group

Patients in the control group received routine treatment or routine treatment including anti-shock therapy, anti-infection therapy, nutritional strengthening, wound treatment, rehydration, and related symptomatic treatment.

### 3.3 Types of outcome measures

#### 3.3.1 Primary outcomes

Primary outcomes included the following:

(1) the wound healing time,
(2) the wound healing rate,
(3) the visual analog scale (VAS) score, and
(4) the scar index.

#### 3.3.2 Secondary outcomes

Secondary outcomes included the following:

(1) the prothrombin time;
(2) the blood endotoxin level;
(3) the interleukin-6 (IL-6) level;
(4) the tumor necrosis factor-α (TNF-α) level;
(5) the incidence of systemic inflammatory response syndrome (SIRS);
(6) cardiac function: creatine kinase (CK), liver function: alanine aminotransferase (ALT) and renal function: creatinine (Cr) levels;
(7) the incidence of multiple organ dysfunction syndrome (MODS);
(8) hospitalization time; and
(9) hospitalization expenses.

### 3.4 Search methods for the identification of studies

#### 3.4.1 Electronic searches

A total of 7 databases were searched by computer, including PubMed, Embase, the Cochrane Library, the China Biomedical Literature Database (SinoMed), the Chongqing Weipu Chinese Science and Technology Journal Full-text Database (VIP), the Tsinghua Tongfang Database (CNKI) and the Wanfang Database. Randomized controlled trials involving the clinical efficacy and adverse reactions of bFGF in the treatment of burns were collected. The search language was not restricted, and the retrieval time spanned from the establishment of the databases to January 2019. All database searches were based on the combination of subject words and free words and were adjusted according to the specific database. Retrieval strategies were determined by multiple presearches. Chinese search terms included “Jianxingchengxianweixibaoshengzhangyinzi”, “bFGF”, “Beifuji”, “Shaoshang”, and “Tangshang”. English search terms included “Fibroblast Growth Factor 2”, “bFGF”, “Basic Fibroblast Growth Factor”, “Class II Heparin-Binding Growth Factor”, “Heparin-Binding Growth Factor Class II”, “FGF-2”, “Fibroblast Growth Factor-2”, “Fibroblast Growth Factor, Basic”, “Prostate Epithelial Cell Growth Factor”, “Burns”, and “Scald”. These searches were simultaneously supplemented with manual searches of the references included in the literature to supplement research that was omitted. Here is an example of searching the Pubmed, the Cochrane Library and SinoMed databases, the retrieval strategy is shown in Table 1.

**Table 1:**
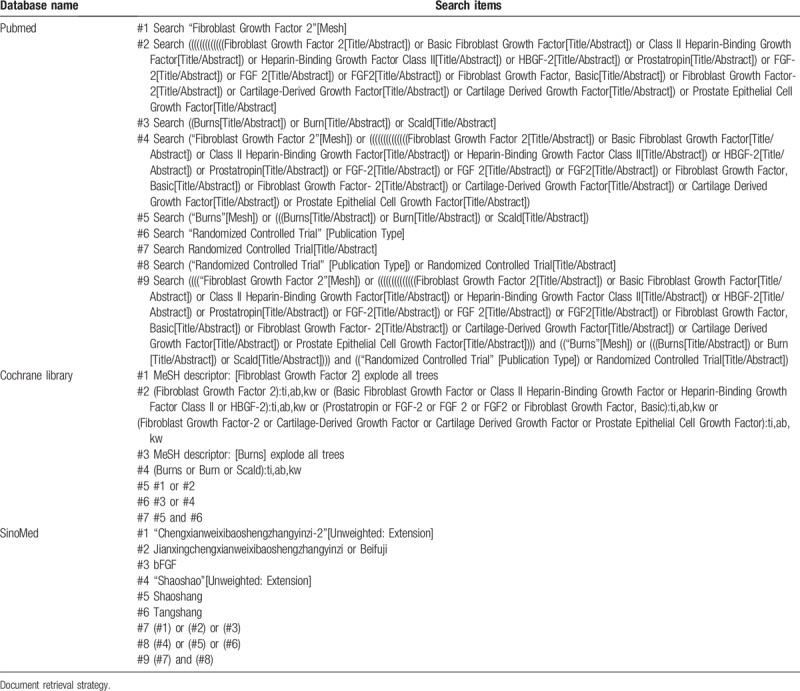
Retrieval strategies of 3 databases: Pubmed, the Cochrane Library, and SinoMed.

#### 3.4.2 Searching other resources

A manual search for references in the selected literature, supplements and special editions of journals without electronic versions was conducted.

### 3.5 Data collection and analysis

#### 3.5.1 Selection of studies

First, according to the previously described inclusion and exclusion criteria, 2 researchers independently screened and extracted the literature.

##### 3.5.1.1 Literature Screening

**Figure 1:**
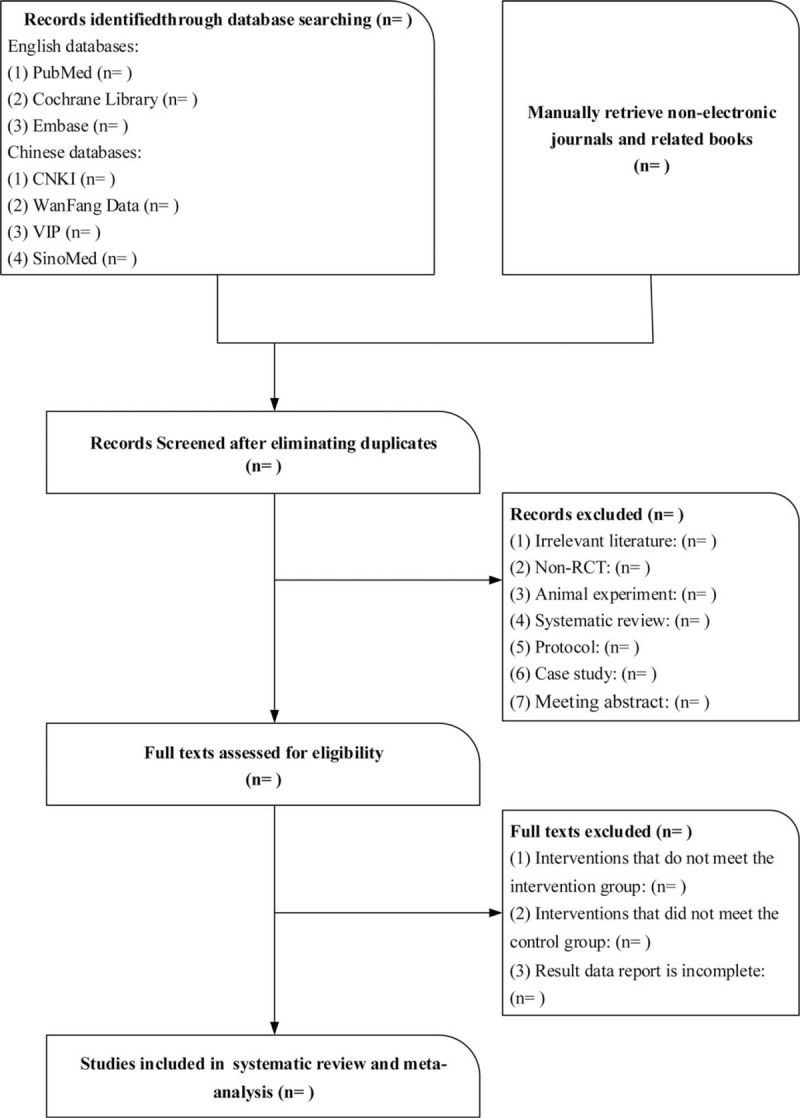
Flow chart of study selection.

First screening: Following searches with the designated Chinese and English search terms, the unqualified documents were removed according to the indicated exclusion criteria. For the secondary screening, the topics and abstracts were read, and summaries, experience summaries, animal model experiments, medical records reports, and repetitive literature from the remaining literature after the first screening were excluded. For the third screening, whether a study was included or not was determined by reading through the full text. If the results from the 2 screeners were quite different, the divergent literature between the 2 parties was discussed, or a third researcher was invited to judge whether the literature should have been included or not. See Figure 1.

##### 3.5.1.2 Data extraction

The following data were extracted:

(1) basic information about the literature (number, title, author, publication time, and publication);
(2) types and methodologies of the research trials (whether or not random allocation methods and random allocation schemes were hidden);
(3) the included case characteristics (the average age, sex ratio, and number of cases in the treatment group and the control group);
(4) the intervention measures (the intervention measures for the control group and the treatment group, the type of drug, drug composition, dose, administration mode, and course of treatment);
(5) the outcome indicators (the primary outcomes, secondary outcomes, adverse reactions, and follow-up results);
(6) and the included research quality assessment indicators (randomized controlled allocation method; whether the allocation was hidden; whether blind methods were used; whether there were reports of termination, withdrawal, or loss of interviews; whether there was intentional processing; whether the data outcomes were complete; whether there were selective reporting outcome indicators; and whether there were other biases).

### 3.6 Study quality assessment

Judgment^[11–13]^ on the quality of the studies was carried out by the following 7 parameters according to the risk bias assessment tool from the RCT evaluation manual of the Cochrane Library:

(1) whether a random allocation method was used;
(2) whether a distribution concealment method was used;
(3) whether a blind method was used for research subjects and researchers;
(4) whether a blind method was used for the evaluation of results;
(5) whether the outcome data were complete;
(6) whether there were selective report outcomes; and
(7) whether there were other biases.

Each included study was evaluated according to the criteria as being “high risk”, “low risk”, or “unclear”. When there were disputes about the evaluations, problems raised were solved after being fully discussed.

### 3.7 Statistical analysis

#### 3.7.1 Data synthesis and statistical analysis

Data synthesis and statistical analysis were carried as follows:

(1) Statistical analysis was carried out by using the meta-analysis software Stata 12.0.
(2) For the selection of effect variables, if the indicators used to test the results of the included studies were bicategorized variables, then the efficacy analysis was represented by the relative risk (RR) and expressed by its 95% confidence interval (CI); continuous variables were expressed by their standard mean difference (SMD) and 95% CI.
(3) For heterogeneity testing, a step to test the homogeneity of the statistical results, that is, to test the degree of variation of the various original research results and the homogeneity of the experiments was conducted as well.
(4) For the meta-analysis, according to the results of the heterogeneity test, when *P* ≥.05 and I^2^ <30, the results were considered to be consistent and were analyzed by a fixed effect model (FEM).

When *P* <.05 and I^2^ ≥30 for heterogeneous results, the origin of the heterogeneity, such as the design scheme, control method, measurement method, illness degree, drug dosage, administration method, course of treatment, age, sex, and other factors, was first analyzed; if these parameters were consistent, subgroup analysis was used to calculate the combined statistics, and if heterogeneity still existed after treatment with the above methods, the heterogeneity results were not neglected. If the included study was still clinically significant, the random effect model (REM) was used.

#### 3.7.2 Sensitivity analysis

Sensitivity analysis was carried out to test the stability of the meta-analysis results. By excluding individual studies that may have led to heterogeneity due to their poor methodology, poor quality, small sample size or large proportion, or by combining effect quantities with different effect modes, whether the results were consistent or not was observed. If there was little difference between the 2 results, the sensitivity of the results was low, and the results were considered stable with high reliability.

#### 3.7.3 Subgroup analysis

If the heterogeneity of a certain outcome index was very large, all data were divided into smaller units according to the available data in the selected literature, and then a comparison was carried out within each subgroup. For example, according to different design schemes, research quality, publication date or a subcategory of research subjects, they were divided into subgroups for reanalysis.

### 3.8 Publication bias

Publication bias is easily caused by positive results in similar studies published in academic journals. This situation is difficult to control and leads to publication bias. The funnel plot and Egger test are often used to evaluate the existence and size of publication bias. If is the funnel graph resembles a symmetrical inverted funnel, it indicates no publication bias. If the center of the funnel graph is asymmetric, there may be publication bias. However, a funnel diagram has the disadvantage of subjectivity and the inability to make statistical descriptions. In this study, Stata 12.0 software was used to determine the number of selected studies that had an outcome index greater than or equal to 8, and the relevant indexes were tested by Egger test. In this study, however, Egger test produced a statistical description in which *P* ≥.05 did not indicate significant publication bias while publication bias existed when *P* <.05. Therefore, the stability of the results was verified by the use of a shear complement test.^[14]^

## 4 Discussion

External medication is an indispensable part of burn treatment. Currently, there are a variety of medicines to treat burn injury. However, it is of great importance to choose an effective, convenient and safe medication to promote early wound healing and relieve patients’ pain. In addition, shortening the course of the disease can reduce the risk of wound infection as well.

Fibroblasts, which are derived from mesenchymal cells in the embryonic stage, are the most commonly observed cells in connective tissue. They also exist in the form of fibroblasts, and they can transform each other under certain conditions.^[15]^ Fibroblasts play a decisive role in the repair of skin wounds. Fibroblasts proliferate in large numbers through mitosis. Large amounts of collagen fibers and matrix were synthesized and secreted from 4 to 6 days after burn, forming granulation tissue together with new capillaries and filling the wound tissue defect to create conditions for epidermal cell coverage.

Recently, with the extensive application of bFGF in the field of burn treatment, the mechanism of bFGF has been further studied. There have been some new findings that are described as follows:

(1) bFGF can inhibit the inflammatory reaction of wounds.^[16,17]^ It may accelerate wound repair and protect the matrix.^[18,19]^ The effects of bFGF on various biochemical changes in the inflammatory response at the molecular level can inhibit the proliferation of lymphocytes by IL-1 and IL-2, the generation of cytokines, the expression of antigen-like enhancers that are enhanced by interferon, and the expression of activator B of the complement bypass pathway induced by cytokines.^[20]^ The alleviation of inflammation can protect burn wounds and reduce wound sepsis and SIRS. However, there are few studies in this field, and these results need further confirmation. By blocking the expression of VEGF, bFGF did not induce the formation of blood vessels by vascular endothelial cells to block the expression of bFGF, which greatly delayed the formation of blood vessels.^[21]^
(2) The presence and expression of bFGF promoted the expression of vascular endothelial growth factor (VEGF), which accelerates angiogenesis and promotes wound healing.^[22]^
(3) The expression of bFGF in deep burn wounds began to appear 6 hours after the burns occurred, and peak bFGF expression presented 1 day after injury and reached a very low level 7 days after the injury. In addition, the external application of bFGF had a significant effect on the activation of endogenous growth factor or the upregulation of growth factor receptor expression.^[23]^

Therefore, it can be concluded from the above results that many studies have reported basic research on the use of bFGF in burn treatment. We also have an understanding of its mechanism and principle in the treatment of burns. In addition, bFGF has been widely used in the clinical treatment of burns. However, there is no systematic report on the efficacy and safety of bFGF in the treatment of burns. Therefore, the aim of this study was to provide evidence-based medical information for the clinical treatment of burns.

## Author contributions

**Conceptualization:** Fan-Jun Meng.

**Data curation:** Da-Chuan Zhan, Yong-Shuai Shen.

**Formal analysis:** Da-Chuan Zhan, Yong-Shuai Shen, Yue-Rong Zhao.

**Methodology:** Da-Chuan Zhan, Yong-Shuai Shen, Yue-Rong Zhao, Fan-Jun Meng.

**Project administration:** Da-Chuan Zhan.

**Software:** Yong-Shuai Shen, Yue-Rong Zhao.

**Supervision:** Fan-Jun Meng.

**Writing – original draft:** Da-Chuan Zhan, Yong-Shuai Shen, Yue-Rong Zhao.

**Writing – review & editing:** Fan-Jun Meng.

Fan-Jun Meng orcid: 0000-0002-2950-3600.
